# Targeted Secretion Inhibitors—Innovative Protein Therapeutics

**DOI:** 10.3390/toxins2122795

**Published:** 2010-12-03

**Authors:** Keith Foster, John Chaddock

**Affiliations:** Syntaxin Ltd, Units 4-10 The Quadrant, Barton Lane, Abingdon, OXON, OX14 3YS, UK; Email: john.chaddock@syntaxin.com

**Keywords:** botulinum neurotoxins, biologics, protein therapeutics, recombinant proteins, genetic engineering, SNARE proteins

## Abstract

Botulinum neurotoxins are highly effective therapeutic products. Their therapeutic success results from highly specific and potent inhibition of neurotransmitter release with a duration of action measured in months. These same properties, however, make the botulinum neurotoxins the most potent acute lethal toxins known. Their toxicity and restricted target cell activity severely limits their clinical utility. Understanding the structure-function relationship of the neurotoxins has enabled the development of recombinant proteins selectively incorporating specific aspects of their pharmacology. The resulting proteins are not neurotoxins, but a new class of biopharmaceuticals, Targeted Secretion Inhibitors (TSI), suitable for the treatment of a wide range of diseases where secretion plays a major role. TSI proteins inhibit secretion for a prolonged period following a single application, making them particularly suited to the treatment of chronic diseases. A TSI for the treatment of chronic pain is in clinical development.

## 1. Introduction

Botulinum neurotoxins (BoNTs) are clinically valuable for treating various neuromuscular and autonomic conditions, and, as clinical products, have sales of more than a billion U.S. dollars [1]. Neurotoxins are clinically effective because they are potent and selective inhibitors of acetylcholine (ACh) release from peripheral nerves, and have a duration of action following a single administration that is often measured in months [2]. The therapeutically useful potency and duration of the neurotoxins’ activity limit their application, however, because they are responsible for the inherent toxicity of the neurotoxins that restrict their therapeutic window. Furthermore, despite targeting a universal mechanism of secretion in eukaryotic cells, the cellular selectivity of the neurotoxins limits their use to treating conditions involving cholinergic nerve activity. There exists, therefore, an opportunity to engineer novel proteins which retain the desirable pharmacological activities of the neurotoxins while reducing their toxicity and broadening their range of therapeutic applications. Recent developments in understanding the structure-function relationships of the neurotoxins, combined with genetic engineering and molecular biology techniques, have made this opportunity a reality. Novel recombinant proteins are now entering the clinic that deliver the benefits of the neurotoxin pharmacology to a wider range of clinical applications without the inherent toxicity of the native neurotoxins. These developments are opening up a new generation of potent biologics for treating chronic diseases.

## 2. Natural History of the Neurotoxins

BoNTs are produced by anaerobic, spore-forming, gram-positive bacteria of the genus *Clostridium*, specifically by several strains of *C. botulinum*, *C. butyricum* and *C. argentinense*. To date, seven immunologically-distinct forms of BoNT have been identified based upon serological cross-reactivity: serotypes A, B, C_1_, D, E, F and G. Within the serotypes, multiple sub-serotypes have been defined based upon sequence homology [3]. A closely related neurotoxin is tetanus toxin (TeNT) produced by *Clostridium tetani*. All BoNTs and TeNT share similar structures and modes of action and are collectively known as clostridial neurotoxins (CNTs) [2]. CNTs are 150 kDa di-chain proteins produced as single-chain polypeptides, and subsequently cleaved by either bacterial or host-organism proteases to form the active, disulfide linked di-chain form of the toxin. 

BoNTs inhibit ACh release from the pre-synaptic terminals of peripheral cholinergic neurons, particularly the motor-end plates of motor neurons. This results in flaccid paralysis. By contrast, TeNT inhibits the release of glycine from inhibitory Renshaw cells in the ventral motor horn of the spinal cord. This results in a dis-inhibition of motor-neuron activity and the characteristic spastic paralysis of tetanus. Despite the contrasting symptoms of botulism and tetanus at the whole animal level, at the cellular and molecular level the CNTs have very similar mechanistic and biochemical actions—inhibition of neurotransmitter release from their respective target neurons [2]. Notably, CNTs are not cyto-lethal toxins. Neither BoNTs nor TeNT kill their target neuron, rather they lead to death of the host via a loss of motor function that results from selective inhibition of neurotransmitter release. Another important aspect of CNT biology is the prolonged, but finite, period of inhibition of neurotransmitter release following intoxication of a neuron. With BoNT, inhibition of ACh release and consequent flaccid paralysis can last from a week to several months depending upon the serotype [4,5]. Similarly, TeNT-induced muscle spasms can last for three to four weeks and then, once the spasms subside, recovery takes several months [6]. With both botulism and tetanus, because of the severity and duration of the disease, people must be treated in a hospital and often require intensive care and respiratory support for several weeks [7,8]. 

The lethal dose of BoNT for humans is not known, but can be estimated from primate studies. The potency varies between serotypes, with BoNT/A being the most potent in humans. By extrapolation, the lethal amounts of crystalline BoNT/A for a 70 kg human would be approximately 0.09–0.15 µg intravenously and 70 µg orally. This makes it the most potent acute lethal toxin known. Given its extreme potency, and routes of intoxication, BoNT is a potential bio-warfare agent and bioterrorist threat agent [7].

## 3. Clinical Use of the Botulinum Neurotoxins

Despite the inherent toxicity of BoNTs and the severe and often fatal nature of botulism, BoNT has become a blockbuster therapeutic and a mainstay for treating many debilitating conditions caused by neuronal hyperactivity. 

### 3.1. History and Background

Christian Andreas Julius Kerner published the first clinical account of a food-borne botulism outbreak in 1817. He extracted the active substance from contaminated food and studied its effects, both in animals and on himself, and in 1822 suggested that the physiological effects of BoNT could be put to therapeutic use [9]. He predicted the toxins’ clinical utility not only in conditions of muscular hypercontraction, but also in autonomic conditions of glandular hyperescretion, such as hyperhidrosis and hypersalivation.

It was more than 150 years, however, before this prophetic proposal was realized. In 1980, Alan Scott of the Smith Kettlewell Eye Research Institute, San Francisco, published a report on the local injection of BoNT/A into ocular muscles to correct strabismus in patients [10,11]. Following this pioneering work, clinicians began to explore how BoNT could be used in the clinical treatment of numerous other conditions, not just muscle hyperactivity. These include conditions of smooth muscle hypercontraction, such as achalasia, and also hyperhidrosis (excessive sweating) and hypersalivation. Thus, since Scott’s original groundbreaking work, the ability of BoNT to paralyze skeletal and autonomic function has been used to clinically treat an increasing range of conditions [1,12,13]. The neurotoxin has also been used for cosmetic purposes, such as treating facial wrinkles like frown lines and brow furrows [14,15].

### 3.2. The Clinical Products

The U.S. Food and Drug Administration (FDA) first approved BoNT/A in 1989 as an orphan drug called “Oculinum”, later marketed by Allergan Inc. as Botox^®^. In 1997, Allergan introduced a new Botox^®^ formulation with reduced ancillary proteins and lower immunogenic potential [16]. In the United Kingdom, a therapeutic formulation of BoNT/A, now marketed as Dysport^®^ by Ipsen Ltd., was developed [17].

Botox^®^ (onabotulinumtoxinA) and Dysport^®^ (abobotulinumtoxinA) consist of a purified neurotoxin complex, which includes the neurotoxin protein and associated non-toxic proteins. The non-toxic proteins are believed to help protect the neurotoxin from the harsh environment of the gut during natural intoxication. Xeomin^®^ (incobotulinumtoxinA) is a recently developed formulation of purified BoNT/A without the non-toxic proteins [18]. A second purified BoNT/A product, PurTox^®^, is in development. Other BoNT/A products, available in parts of Asia, include CBTX-A and Neuronox^®^ [19]. While the most commonly-employed therapeutic BoNT serotype is type A, there is also a BoNT/B product, again a purified complex, marketed as Neurobloc^®^ in Europe and Myobloc^TM^ in the United States [20]. There are no clinically approved products of the other five serotypes, though exploratory pre-clinical studies have investigated some of the characteristics of the protein family, for example with respect to duration of action [21,22,23,24,25]. 

Although, with the exception of Neurobloc^®^/Myobloc^TM^, all the commercially-available BoNT products are type-A based, they are not directly interchangeable [26,27,28,29,30]. The unit of activity for measuring the potency of the different preparations is the mouse LD_50_ (one unit is the amount of intraperitoneally-injected toxin that kills 50% of a group of mice). Due to differences in the method of production, formulation and performance of the LD_50_ assay, the units for a given product are not clinically equivalent to those of another product. It is therefore critically important to recognize that each product requires different unit doses to achieve an equivalent, safe clinical effect. The various BoNT/A products are distinct and unique, and should not be considered as generically equivalent products. 

### 3.3. Neuromuscular Uses

Treatment of muscle hyperactivity continues to be the main use of BoNT and the basis of its regulatory approval. Following Scott’s pioneering studies in strabismus, BoNT/A was shown to relieve the muscle spasm involved in various focal dystonias. In 1987, ophthalmologist Jean Carruthers initiated the cosmetic use of Botox^®^ when she observed during treatment of blepharospasm that vertical glabellar creases (frown lines) disappeared [31]. In addition to dystonia, BoNT injections can treat other movement disorders such as tremors of the hands and head, and also motor dysfunctions associated with Tourette syndrome. 

As well as involuntary movement disorders, BoNT has been used to treat muscle tone disorders, including spasticity associated with cerebral palsy, stroke, brain trauma and multiple sclerosis [32,33,34]. Treatment of leg spasticity in children with cerebral palsy with BoNT means surgical lengthening of the heel cord to improve gait can be avoided [35,36]. Early use of BoNT treatment in these children may also prevent musculoskeletal deformities and other orthopedic problems later in life. Several studies have shown BoNT can improve functionality in spasticity, particularly of the upper limbs, following stroke [32]. When used early, BoNT may prevent development of complications of spasticity such as contractures. Following BoNT therapy, relief of the rigidity associated with various parkinsonian disorders and stiff person syndrome has also been reported [32]. 

### 3.4. Autonomic and other Non-Neuromuscular Uses

Several studies have reported that following BoNT application to treat muscle spasm, associated pain is relieved [27,37]. Among the musculoskeletal and muscle spasm associated pain disorders reported to benefit from BoNT injection are low back pain, fibromyalgia-myofascial pain, temporomandibular joint and orofacial pain [27]. There have also been several studies reporting BoNT can treat muscle contraction headaches (chronic daily, tension or cervicogenic headaches) and migraines [27]. Allergan Inc. have progressed Phase III studies and have received approval from the U.K. Medicines and Healthcare products Regulatory Agency (MHRA) of Botox^®^ as a preventive treatment for chronic migraine headaches. Data supporting the clinical studies have been reported [38]. The FDA is soon to announce whether the product is approved for use in the United States. Interestingly, the pain relief following BoNT administration tends to start earlier and end later than the muscle relaxation. This has led to the conclusion that the pain-relieving activity of BoNT is a result of mechanisms other than direct muscle relaxation, and several mechanisms have been proposed. These include a direct effect on the release of nociceptive neurotransmitters from the peripheral terminals of sensory nerves [39,40], thereby reducing local neurogenic inflammation and pain [41]. Alterations in the firing activity of supra-spinal afferent projections from muscle spindle fibres could also affect central sensory processing [42]. Whatever the explanation, the potential for BoNT to relieve certain pain conditions associated with muscle spasm continues to be actively investigated [27,43]. 

Given that BoNT inhibits ACh release at cholinergic parasympathetic and post-ganglionic sympathetic nerves, it is an effective treatment for several disorders of the autonomic nervous system [29,43,44]. Intradermal injection of BoNT is a highly effective treatment for focal hyperhidrosis. Whilst for the majority of applications the clinical benefit of BoNT/A is of the order of three months, in hyperhidrosis the benefit has been reported to last up to three years [44]. Other autonomic applications of BoNT include treating hypersalivation associated with Parkinson’s disease, hyperlacrimation and Frey’s syndrome.

The ability of BoNT injections to relax or inhibit smooth muscle contractility and treat several gastrointestinal disorders has also been investigated [45]. Dysphagia, achalasia and other esophageal spasms have all been successfully treated with local BoNT injections, as has spasm of the sphincter of Oddi, spasm of the rectal sphincter and chronic anal fissure. There have also been several interesting, recent reports that injecting BoNT/A into the gastric antrum using endoscopy can treat obesity [46]. In laparatomized rats, gastric injection of BoNT/A was reported to significantly reduce their food intake. The human clinical studies reported to date, however, have yielded conflicting results. This, therefore, remains an interesting potential area for therapeutic use of BoNT.

Currently, one of the most active and exciting areas of development for the clinical utility of BoNT outside of musculoskeletal applications is in urology [47]. Patients with neurogenic bladder suffer from detrusor overactivity, which may be combined with detrusor sphincter dyssynergia. Both conditions lead to high intravesical pressure and incontinence. In patients with detrusor overactivity, BoNT injection at perhaps 20 to 30 sites in the detrusor muscle under cystoscopic guidance has been shown in several studies to improve continence, void volume, urinary frequency and bladder capacity. BoNT has also been reported to have led to benefit in patients suffering from detrusor sphincter dyssynergia following spinal cord injury. In its recent assessment of BoNT in the treatment of autonomic disorders, the American Academy of Neurology concluded that: “BoNT should be offered as a treatment option for the treatment of … detrusor overactivity … [and] … detrusor sphincter dyssynergia after spinal cord injury” [43].

## 4. Newer Applications and Future Developments

There has been a huge increase in the range of medical disorders for which therapeutic use of BoNT has been explored and demonstrated since 1980, and there continue to be reports of new applications [1,29,48]. The clinical utility of this remarkable agent is expected to continue to expand for the foreseeable future. One area that will be important in the future will be to properly assess the comparative clinical effectiveness of the various BoNT products available and to assess the effect and role of the ancillary proteins on the clinical properties of the products [19,29]. In recent years, it has become apparent that, within serotypes, there are multiple sub-serotypes that differ in biochemical properties [3,49,50]. These sub-serotypes may provide different therapeutic opportunities from current products. All of the commercially available BoNT/A products are based upon sub-type A1. Recent preliminary clinical studies with a type A2 neurotoxin may indicate clinically meaningful differences from the A1 products [51]. 

Improvements in the understanding of the toxin serotypes and subtypes promise to further expand the clinical value of BoNT as a therapeutic agent. They will not, however, address fundamental limitations inherent in the neurotoxin. These limitations derive from the properties that make BoNT such a successful product, namely its potent and prolonged inhibition of peripheral cholinergic neurotransmission [2]. The potency of neurotoxins results from their high affinity selective binding to specific receptors on their target neurons. As a result of this selectivity, the unique ability of CNT, including BoNTs, to inhibit secretion cannot be applied to non-neuronal targets [2]. The potency and duration of effect of the neurotoxins also means that they have a very narrow therapeutic window and can be severely toxic if incorrectly used [52]. There have been reports of severe and even fatal outcomes of incorrect administration of BoNT products, and the FDA has recently required a black box warning to be included in licensed BoNT products [53]. There is, therefore, a tremendous opportunity to develop novel biologics that retain the therapeutically successful pharmacological properties of the BoNT products, but which have improved profiles in regard to both therapeutic window and breadth of therapeutic opportunity. Recent developments in the understanding of the detailed mechanism of neurotoxin function coupled to an understanding of the structural basis of that activity have made that opportunity a reality. 

## 5. Molecular Basis of Neurotoxin Action

### 5.1. SNARE Cleavage and Role of SNARE Proteins

Inhibition of neurotransmitter release by the CNTs is driven by the proteolytic cleavage of SNARE (soluble N-ethylmaleimide-sensitive factor attachment protein receptor) proteins [2]. The SNARE proteins are a large superfamily of proteins that are key to membrane fusion in eukaryotes [54,55,56]. Specific SNARE proteins present on two opposing membranes interact to form a stable SNARE complex that leads to membrane fusion. Formation of a SNARE complex is sufficient to drive membrane fusion [57,58,59]. The CNTs are Zn^2+^-dependent metallo-proteases that have evolved to selectively cleave SNARE proteins involved in the docking and fusion of synaptic vesicles with the pre-synaptic membrane in their target neurons. Each CNT cleaves one or other of the SNARE proteins at a single peptide bond [2]. The exact identity of the SNARE protein and the peptide bond that is cleaved varies between CNTs, but in each case cleavage prevents formation of a productive SNARE complex and vesicle fusion, and thereby inhibits neurotransmitter release.

Although the SNARE proteins and their role in secretion were first identified in neuronal cells, the SNARE complex is not restricted to nerve cells [60]. It is a universal mechanism underpinning secretion from all cell types. In fact, secretion is not the only SNARE protein-dependent vesicle fusion event at the cell membrane. The transport of a wide number of integral membrane proteins to the cell surface is mediated via SNARE protein-dependent vesicle fusion. In particular, the regulated expression of many receptors, ion channels and transport proteins involves SNARE-dependent fusion of intracellular vesicles [55]. Examples include the insulin-regulated expression of the glucose transporter Glut4 in adipocytes [61] and the up-regulation of the ion channel TRPV1 in sensory neurons [62,63], both of which have been shown to be sensitive to SNARE protein cleavage by endopeptidases from CNTs. CNT endopeptidases therefore can affect many vesicular transport activities within different cells. The reason that CNTs do not affect these events in non-nerve cell types is because they have evolved to specifically bind to nerve cells. 

### 5.2. Molecular Basis of Neurotoxin Action

The di-chain CNT protein consists of a light chain (LC) of approximately 50 kDa and a heavy chain (HC) of approximately 100 kDa [64]. The LC is a Zn^2+^-dependent metallo-protease responsible for the SNARE cleavage by the neurotoxin. In order to access its substrate protein and cleave the SNARE protein, the LC has to gain entry into the cytosolic compartment of the pre-synaptic nerve terminal. The HC is effectively a delivery vector that binds with high affinity to receptors on the pre-synaptic membrane and subsequently enables delivery of the LC into the neuronal cytosol. 

The binding event underpins both the highly selective neuronal targeting and the potency of the neurotoxins. A dual receptor model of interaction has been proposed, in which the neurotoxins initially interact with polysialogangliosides, particularly GD1b and GT1b, at the pre-synaptic terminal [65]. The neurotoxin then binds with high affinity to a protein receptor that leads to internalization into an intra-cellular vesicular compartment. For BoNT/A, the protein receptor has been identified as an intra-lumenal domain of the synaptic vesicle protein SV2 [66]. Glycosylated forms of SV2A and SV2B have been identified as the protein receptors for BoNT/E [67] and recently glycosylated SV2 has also been reported to be the receptor for BoNT/F [68]. A lumenal domain of the synaptic vesicle protein synaptotagmin I or II has been identified as the protein receptor for both BoNT/B [69,70] and /G [71,72,73]. The identity of the protein receptor has not yet been determined for the other CNTs. The fact that the receptors identified to date are lumenal domains of synaptic proteins is consistent with the property of neurotoxins that they are more effective at inhibiting active neurons [74,75,76,77,78]. 

Following acidification, the H_N_ domain of HC forms a pore in the vesicle membrane, allowing entry of the LC into the cytosol [79]. A key feature of both botulism and clinical neurotoxin therapy is a long duration of inhibition of neurotransmitter release [22]. Although the different serotypes vary considerably in the duration of this effect, all inhibit neurotransmitter release from several days to weeks following a single administration. Whilst the exact molecular basis of this prolonged duration and the explanation for the differences observed between serotypes is still an area of active investigation [64], survival of the LC [5,80] and its SNARE protein cleavage products [81,82] within the nerve terminal are key factors.

The first reported crystal structure for a CNT was for BoNT/A [83]. Subsequently, the structures of a number of other CNTs, or fragments thereof, have been reported (Table 1). These structures show that, despite considerable heterogeneity at the primary sequence level, the different serotypes display a highly-conserved multi-domain tertiary structure [64]. To date, the most dissimilar structure reported is that for BoNT/E, which, while displaying the same domains as identified in the other CNT structures, has a distinct spatial arrangement of the domains within the overall global fold of the protein [84,85]. The solution of the tertiary structure of neurotoxin protein has enabled a very close correlation of structure to function for the various domains. 

**Table 1:** CNT structures in the Research Collaboratory for Structural Bioinformatics protein databank.

Domain/Fragment	Serotypes studied
**BoNT**	A, B, E
**LC**	A, B, C, D, E, F, G, TeNT
**HC or H_C_**	A, B, C_1_, D, F, G, TeNT
**LH_N_**	A, B

The HC consists of two distinct domains: the carboxy terminal half, H_C_, and the amino-terminal half, H_N_. H_C_ further comprises two sub-domains, each of approximately 25 kDa: the extreme carboxy-sub-domain, H_CC_, and the H_CN_ sub-domain. The H_CC_ sub-domain is where the ganglioside and protein binding sites are located [64]. The precise function of the H_CN_ sub-domain is currently unknown, although it may help orientate the binding domain relative to the translocation domain and enable efficient insertion of the translocation domain [84]. In BoNT/A and /B, the binding and catalytic domains flank the translocation domain and there is no interaction between them. In BoNT/E, by contrast, the binding and catalytic domains are on the same side of the translocation domain and all of the domains interact, resulting in a tight globular structure [84]. The arrangement of the domains in the BoNT/E structure may represent a translocation competent conformation enabling more rapid entry of BoNT/E LC, explaining the faster toxic rate of BoNT/E relative to other serotypes [84].

The H_N_ domain is the translocation domain and consists of two long α-helical regions. Acidification of the endosomal compartment following internalization of the bound toxin triggers the H_N_ domain to undergo a conformational change enabling insertion into the vesicle membrane [86]. An unusual feature of the H_N_ domain is a long loop, aptly referred to as the belt, which wraps around the LC domain. It has been proposed that this belt region acts as a surrogate pseudosubstrate LC inhibitor, protecting the active site and acting as a chaperone during the translocation process [79,87]. The H_N_ channel of BoNT/A has an estimated diameter of 15 Å, which is too small for the LC to pass through without unfolding, and there is evidence that under the low pH conditions prevailing in the endosome, the LC adopts a molten globule-like structure [88] and translocation occurs via a partially-unfolded LC conformation [79]. Translocation of the LC into the cytosol via the H_N_ channel enables the protease to access its substrate SNARE protein.

The LC itself comprises a mixture of α-helices, β-sheets and strands that form a compact globular-like protein structure. There is a large, open cavity, which contains the zinc atom, coordinated by two histidine residues (229 and 233), a glutamate residue (267), and a water-mediated coordination through an additional glutamic acid. This region shares structural similarity to the active site of Zn^2+^-metallo-proteases, such as thermolysin, and contains the characteristic HExxH zinc protease consensus motif [2]. Unlike other zinc-dependent proteases, CNT LCs need an extended substrate sequence for optimal catalytic activity [89,90]. These include substrate-binding sites remote from the scissile bond, and these exosites presumably align the substrate at the catalytic site and are responsible for the exquisite substrate selectivity of the proteases [91,92,93].

## 6. Recombinant Engineering and Application of Neurotoxin Pharmacology

Understanding CNT structure and its relation to function has opened up the possibility of developing recombinant proteins based on the specific pharmacology of the different domains [94]. The investment in the basic science of CNT biology over many years provides the potential for the design and creation of therapeutic proteins that deliver selected aspects of the neurotoxins’ unique pharmacology without the toxicity. The ability to engineer recombinant proteins incorporating specific components of the neurotoxins is further aided by the linear organization of the discrete functional domains within the neurotoxin gene which enables independent manipulation [95]. Harnessing the inherent functional domains of the CNTs both overcomes the limitations of the current neurotoxin products and opens up new clinical opportunities. 

### 6.1. Re-Targeting of Neurotoxin Protease: The Rationale Behind TSIs

**Figure 1:**
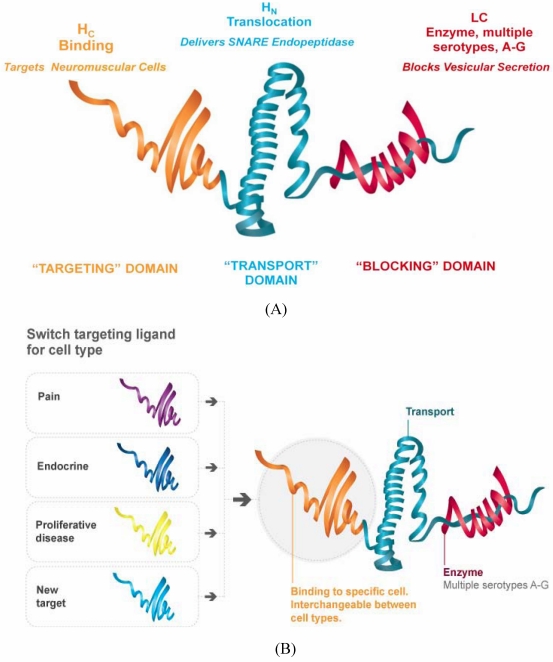
Schematic representation of Botulinum neurotoxin structure in relation to engineered Targeted Secretion Inhibitor (TSI) proteins. (A) Botulinum neurotoxin domain structure; (B) TSI engineering.

The neurotoxin LC is a highly specific endopeptidase that has evolved to selectively cleave the SNARE proteins involved in synaptic vesicle docking and fusion with the pre-synaptic membrane. Given, however, that SNARE proteins represent a universal mechanism of vesicle fusion in eukaryotic cells [60], underpinning both secretion and the transport of integral membrane proteins to the cell surface, the potential exists that neurotoxin LC could cleave SNARE proteins and block vesicle fusion events in a variety of cells. Indeed it has been known for many years that, whilst neurotoxins naturally only enter neurons to cleave SNARE proteins and block secretion, artificial introduction of the neurotoxin, or LC, by microinjection, permeabilization or transfection can affect vesicular trafficking in a wide variety of cell types [2]. Delivering the endopeptidase domain to cells not targeted naturally by CNTs, for example, non-neuronal cells, is therefore a very exciting way of generating more widely applicable therapeutics employing the unique pharmacology of the neurotoxin. Targeted Secretion Inhibitors (TSIs) are novel engineered proteins that extend the utility of the CNT LC into a wide spectrum of cell types. These proteins incorporate the LC endopeptidase domain and H_N_ translocation domain to enable cytosolic delivery, the LH_N_ fragment, together with a binding domain that binds to a cell surface receptor on the proposed target cell (Figure 1). By targeting membrane receptors or cell surface proteins that internalize into the cell, the H_N_ domain is able to enter a suitable endosomal compartment and form a translocation pore thereby enabling entry of the LC into the cytosol. In addition to allowing the potent pharmacology of SNARE protein cleavage to be applied to non-neuronal cells, altering the binding domain also enables creation of neuronally targeted therapeutics with improved neuronal selectivity and an enhanced therapeutic window relative to native BoNTs.

Botulinum neurotoxins have a very clear structure-function relationship as indicated diagrammatically in Figure 1A, where the three discrete domains identified within the crystal structure are represented in different colors and their respective functions provided. In TSI proteins, the receptor binding domain of the neurotoxin is replaced by a novel protein or peptide binding domain that defines new target cell specificity; this is represented diagrammatically in Figure 1B. The engineered TSI proteins are created by designing and synthesizing a recombinant gene encoding the desired protein and then expressing the gene in a suitable expression host, typically *E. coli*.

### 6.2. Re-Targeting of Neurotoxin Protease: Development of Recombinant Platform

Recombinant expression of a catalytically-active, stable LH_N_ fragment of BoNT/A was first reported in 2002 [96]. Subsequently, the expression and purification of catalytically active LH_N_/B and LH_N_/C from *E. coli* was also reported [97]. The recombinantly expressed LH_N_ proteins were fully functional following proteolytic activation to generate the di-chain protein. In all cases, the recombinant LH_N_ fragments had very low toxicity, and in the case of recombinant LH_N_/A, they were less toxic than a highly-purified LH_N_/A prepared by proteolytic treatment of BoNT/A. The LH_N_ fragment is effectively non-toxic because it lacks the necessary H_C_ domain with which to bind to acceptors on the neuronal surface. The crystal structure for recombinant LH_N_/A has recently been reported [98] and compared to that for BoNT/A. The recombinantly expressed fragment was structurally equivalent to the relevant domains within the intact native BoNT. Recombinant LH_N_/B also retains the crystal structure of the equivalent domains in the intact BoNT/B protein [99]. Thus, recombinantly expressed LH_N_ proteins retain both the functionality and structure of the relevant domains from the parent neurotoxin.

A fully recombinant fusion protein consisting of the LH_N_-fragment of BoNT/C_1_ and epidermal growth factor (EGF) has been reported [100]. The protein was expressed as single chain polypeptide with a specified enzyme cleavage site between the endopeptidase and the remainder of the polypeptide. This enabled selective activation of the expressed protein using a defined exogenous protease to produce the active di-chain protein. The potential exists for the linker region containing this activation site to be varied to optimize activation of the particular protein and spacer regions can be incorporated to optimize the spacing of the various components within the engineered protein. This protein represents a prototype example of a TSI protein incorporating relevant components of a clostridial neurotoxin, but with modified cell targeting properties allowing targeted delivery of the LC into a novel, specified target cell determined by the engineered binding domain. 

### 6.3. Re-Targeting of Neurotoxin Protease: Proof-of-Concept

The first description of modifying a CNT’s cell-binding domain was by Bizzini, who, as part of a study into the mechanism of tetanus toxicity, designed conjugates coupling either ricin toxin B-chain or wheat germ agglutinin to a proteolytically generated fragment of TeNT that retained functional LC and H_N_ domains of the neurotoxin [101]. The first retargeted BoNT reported was a conjugate of nerve growth factor and LH_N_/A that was able to deliver the type A endopeptidase into PC12 cells resulting in cleavage of SNAP-25 and inhibition of noradrenaline release [102]. Following this, a conjugate of wheat germ agglutinin and the LH_N_/A fragment was reported to deliver the endopeptidase into both neuronal and non-neuronal cell types with a consequent cleavage of SNAP-25 and inhibition of secretion [103]. One of the cell lines studied was the hamster pancreatic β cell, HIT-T15, where a significant concentration-dependent inhibition of stimulated insulin release was found to correlate with cleavage of SNAP-25. HIT-T15 cells are resistant to the effects of BoNT/A, so this result demonstrated that it is possible to internalize the endopeptidase into the cytosol of a cell normally resistant to the effect of BoNT, thus confirming the ability of the H_N_ domain to function in the new target cell following binding and endocytosis via the new binding domain.

An exciting development of this approach was targeting a conjugate of *Erythrina cristagalli* lectin and LH_N_/A (ECL-LH_N_/A) to nociceptive afferents, a therapeutically-relevant target cell, via galactose-containing carbohydrates specific to those particular neurons [104]. ECL-LH_N_/A inhibited release of both substance P and glutamate from embryonic dorsal root ganglion neurons in culture. Importantly, no effect was seen at equivalent concentrations on cultures of embryonic spinal cord neurons from an anatomically adjacent region, thereby demonstrating selectivity of action between neuronal populations [104]. The effect in embryonic dorsal root ganglion neurons was maintained for at least 25 days following a single treatment, demonstrating that the conjugate had retained the duration of effect of the native BoNT. Intrathecally-administered ECL-LH_N_/A significantly reduced the nociceptive inputs to convergent dorsal horn neurons by primary sensory afferents of the C-fiber and Aδ types, whereas there was little or no effect on sensory inputs from Aβ-fibers [104]. Intrathecal ECL-LH_N_/A also resulted in prolonged withdrawal latency in a ‘hotplate’ model of acute thermal pain. This effect was sustained for more than 30 days post-administration of the conjugate, confirming the *in vitro* finding that the conjugate retained the duration of effect properties associated with the native neurotoxin [105].

Another approach to generating TSI in which the targeting properties of CNT are modified in order to create proteins with differentiated properties and therapeutic potential from native neurotoxins is the creation of hybrids between CNTs that combine the properties of the component domains. Delivery of TeNT LC to peripheral motor-neurones using BoNT HC has been reported, resulting in a spastic paralysis reminiscent of botulism [106]. The creation of chimeras of BoNT/A and /E in which the H_C_ domain of one serotype was expressed recombinantly fused to the LH_N_ of the other serotype have also been reported [107]. The translocation properties of the hybrid were clearly differentiated, and reflected those of the parent LH_N_ domains, whilst the neuronal specificity was influenced by the identity of the H_C_. The therapeutic potential of creating a hybrid neurotoxin TSI in this manner was demonstrated by showing that the LH_N_/E-H_C_/A hybrid protein was effective at blocking the responsiveness of nociceptive neurons to inflammatory or pain stimuli, whereas BoNT/A was ineffective [108].

With the structural definition of the binding loops within the CNT H_C_ domain and the availability of co-crystal structures for many of the interactions with both ganglioside and protein receptors, it has been possible to begin to define key residues involved in those interactions [64]. For example, a ganglioside binding cavity within the H_CC_ domain of BoNT/A and /B defined by the conserved motif H…SXWY…G has been identified [109]. Site directed mutations within this site modified both the ganglioside binding affinity and the toxicity of the neurotoxin. Whilst many of the mutants displayed reduced binding and toxicity, some displayed an enhanced activity, with the most potent displaying a three-fold increased toxicity relative to wild type toxin [110]. This begins to provide a rational basis for engineering mutated CNT with modified, particularly enhanced, potency as improved clinical products. This would enable reduced dosing and thereby potentially reduce the risk of patients developing neutralizing antibodies. Understanding the nature of the binding interactions and identification of the binding motifs and key residues also potentially allows the specificity of the binding interaction to be modified, although changing the selectivity of the binding event by modifying the binding sites has yet to be demonstrated.

In addition to engineering the binding domains of CNT to modify functionality and enable creation of designed TSI proteins, attention has also been given to the endopeptidase domain and its substrate specificity. A major limitation to using clostridial endopeptidases to cleave SNARE proteins and thereby inhibit vesicle trafficking in non-neuronal cells is in relation to the SNAP-25 cleaving serotypes, BoNT/A, /C and /E. SNAP-25 is restricted in its expression to neurons and the ubiquitously expressed homologue, SNAP-23 [111], is not, in man, a substrate for any of the relevant serotypes [112]. This means that retargeting the LC from serotypes /A, /C or /E, will not impact vesicular trafficking in non-neuronal cells. This limitation to the applicability of clostridial endopeptidase has recently been challenged by the reported engineering of a mutated BoNT/E LC that can cleave human SNAP-23 [113]. The potential to deliver a mutated LC of serotype E and impact SNAP-23 mediated vesicle trafficking is therefore a real possibility, further expanding the opportunity represented by TSI to provide novel therapeutic proteins for treatment of chronic diseases not amenable to treatment with natural CNT.

The potential to create novel proteins that enable delivery of the endopeptidase component of a CNT to a diverse range of cell types is now well established. The ability of such proteins to produce pharmacological effects in disease-relevant animal models, has also established the therapeutic potential of this approach. The various studies have demonstrated that the retargeted endopeptidase proteins retain the prolonged duration of action that is the hallmark of CNTs. This means that recombinant proteins based upon this approach are particularly suitable for treating chronic diseases.

### 6.4. Clinical Opportunities for TSIs

In terms of pre-clinical and clinical exemplification of the TSI platform, a novel targeted BOTOX has recently been reported to have completed Phase I studies and to have entered Phase II studies in Post-herpetic neuralgia (PHN) [114]. By targeting C fibers and inhibiting release of nociceptive neurotransmitter through cleavage of SNARE proteins, it is anticipated that new treatments for chronic pain will be developed that harness the extended duration of action of both native neurotoxin and future targeted toxin products. It is also reported that a trial of the same targeted BOTOX that is being assessed in PHN has also commenced in idiopathic overactive bladder [115]. It is therefore to be anticipated that in the foreseeable future, data will begin to emerge that will demonstrate the reality of the clinical promise offered by TSI proteins based upon the endopeptidase activity of CNT. A list of just some of the potential applications of TSI proteins is illustrated in Table 2. 

**Table 2:** A selection of potential applications of TSIs.

Neuronal targeting	Non-neuronal targeting
Pain	Endocrine diseases
Idiopathic Overactive Bladder	Inflammation
Neuropathic Overactive Bladder	Mucus hypersecretion
	Cancer

With such broad clinical potential, TSIs are an exciting application of innovative science. As an innovative platform, however, it is inevitable that not all of the answers to classic drug development questions are yet established in the literature. For example, what is the bioavailability, how stable are the fusion proteins, how well do they reach their specific target *in vivo*? Pre-clinical observations indicate that the receptor binding domain engineered into TSIs is capable of directing the novel protein to the target cell of choice and effecting inhibition of secretory processes. As TSI proteins enter clinical trials in man data will begin to become available that will address tolerability and safety while defining the dose interval and minimal effective clinical dose for clinical efficacy for the TSI platform. With the emergence of this information, together with data from further pre-clinical studies spanning a range of indications, many of these important ‘drugability’ questions will begin to be answered for this novel therapeutic platform. 

## 7. Conclusions

BoNT/A is a major therapeutic product that can be widely used to treat various neurological and neuromuscular conditions. The therapeutic success of the BoNTs results from their specific and potent inhibition of neurotransmitter release from peripheral cholinergic neurons combined with a duration of action measured in months. The clinical utility of the neurotoxins is, however, severely constrained, both by their limited range of target cells and narrow therapeutic window. Advances over the last twenty years in understanding the structure and biology of CNTs, combined with developments in recombinant protein engineering is opening up opportunities to engineer novel therapeutic proteins based upon the unique pharmacological properties of the CNTs. One such opportunity, Targeted Secretion Inhibitors, is already progressing in the clinical setting and pre-clinically for a range of indications not treatable with neurotoxin products. These proteins will increase the medical benefits achieved through clinical application of the native neurotoxins, particularly BoNT/A, while removing the inherent toxicity and providing proteins with a much-improved therapeutic window. Harnessing the properties of the neurotoxins’ protein domains in novel recombinant proteins will lead to the creation of a completely new class of biologics. By inhibiting secretion, these will treat chronic conditions like chronic pain, which currently have few effective treatments.
